# Trends in concurrent tobacco use and heavy drinking among individuals 15 years and older in Mongolia

**DOI:** 10.1038/s41598-022-21094-7

**Published:** 2022-10-05

**Authors:** Supa Pengpid, Karl Peltzer

**Affiliations:** 1 Department of Health Education and Behavioral Sciences, Faculty of Public Health, Mahidol University, Bangkok, Thailand; 2 Department of Public Health, Sefako Makgatho Health Sciences University, Pretoria, South Africa; 3 Department of Psychology, College of Medical and Health Science, Asia University, Taichung, Taiwan

**Keywords:** Psychology, Risk factors

## Abstract

The study aimed to evaluate trends in the prevalence and correlates of current tobacco use only (= CT), current heavy drinking only (= CHD), and current tobacco use and current heavy drinking (= CTHD) in people 15 years and older from 2009 to 2019 in Mongolia. Cross-sectional data were analyzed from 5,438 individuals (15–64 years) of the Mongolia STEPS 2009 survey, 6,013 (15–69 years) of the Mongolia STEPS 2013 survey, and 6,654 persons (15–69 years) of the Mongolia STEPS 2019 survey and responded to questions about substance use, other health risk behaviours and physical measurements. Trend analysis shows that the prevalence of CT increased from 14.4% in 2009 to 15.3% in 2019, and the prevalence of CHD significantly decreased from 13.3% in 2009 to 10.3% in 2019, and the prevalence of CTHD significantly decreased from 14.2% in 2009 to 9.4% in 2019. Middle and older age, male sex (13 times higher for CT, 3.4 times higher for CHD, and 28 times higher for CTHD) and high physical activity significantly increased the odds of CT, CHD and CTHD. Higher education decreased the odds of CT but increased the odds of CHD. Urban residence was positively associated with CT, and among women with CTHD. Underweight decreased the odds of CT, CHD, and CTHD, and obesity decreased the odds of CT and CTHD and increased the odds of CHD. Hypertension was positively associated with CHD and CTHD, while sedentary behaviour was positively associated with CT and CTHD. Being Khalkh by ethnicity increased the odds of CT and inadequate fruit and vegetable intake increased the odds of CHD. More than one in six persons 15 years and older engaged in CT and more than one in ten engaged in CHD and CTHD. Several sociodemographic factors, such as male sex and middle and/or older age, and health variables, such as obesity and hypertension for CHD, were identified associated with CT, CHD, and CTHD that can help in guiding public interventions.

## Introduction

Concurrent heavy alcohol use and tobacco use is an increasing public health concern globally^1,2^, and can be defined as using tobacco and alcohol during the past 4 weeks, and not simultanously. However, there is little information on the epidemiology and associated factors of concurrent heavy alcohol use and tobacco use, particularly in low and middle-income countries, such as Mongolia, which reduces our ability to design effective interventions^3^. In Mongolia, approximately 30% of women and 50% of men are current drinkers, and most believe that heavy episodic drinking is common in Mongolia^4^. According to World Health Organization^5^ estimates in 2016, the prevalence of alcohol use disorder in both sexes was 7.8% (13.3% in men and 2.5% in women) and the proportion of heavy episodic drinkers (“consumed at least 60 g or more of pure alcohol on at least one occasion in the last 30 day”) is 16.4%, 27.9% in men and 5.3% in women) in Mongolia. Almost half of men (46.3%) and 6.8% of women in Mongolia were smokers in 2010^6^. In a national survey in 2018 in Mongolia, the prevalence of current tobacco use was 9.2% among women (15–49 years) and 58.0% among men (15–49 years), and the prevalence of current alcohol use was 27.4% and 47.2% among women and men, respectively^7^. The estimated age-standardized prevalence of current tobacco use among those aged 15 years or older in Mongolia was 29.8% in both sexes in 2019^8^. Among men in Mongolia, the highest increase in the age-standardised death rate between 1990 and 2019 occurred in alcohol use disorders (628.6%)^9^.

Morbidity and mortality risk is significantly higher in concurrent current tobacco users and current heavy alcohol users (= CTHD) than in the use of current heavy drinking only (= CHD) or current tobacco use only (= CT)^10,11^. Reasons for the higher risk in CTHD include multiple factors, including genetic, behavioural and pharmacological^12,13^. For instance, people who engage in CTHD have a greater risk of specific cancers than those who use CT or CHD^14^. Biological mechanisms for explaining the higher risk in CTHD include that “both alcohol and nicotine act on a brain system called the mesolimbic dopamine system, which mediates the rewarding and reinforcing properties of both drugs. Modification of the activities of the mesolimbic dopamine system can interfere with the effects of both alcohol and nicotine. Another mechanism that may contribute to alcohol-nicotine interactions is cross-tolerance to the effects of both drugs. Finally, genetic studies in humans and of selectively bred mouse and rat strains suggest that shared genetic factors help determine a person's liability to use or abuse both alcohol and nicotine.”^15^.

There is little information on concurrent alcohol and tobacco use in Asian countries. In Thailand, the prevalence of concurrent daily smoking and hazardous or harmful alcohol use was 5.2%^16^, and in another study in Thailand, the prevalence of co-use of current smoking and alcohol consumption during the last 12 months was 15.2%, tobacco use only 4.7% and alcohol use only 18.9%^17^. In Laos, the prevalence of CTHD was 18.6%, CT 15.2% and CHD 28.4%^18^. In Korea, the prevalence of currently smoking and drinking monthly was 13.1%, currently smoking only 4.2%, and drinking monthly only 38.7%^19^. In Sri Lanka, the prevalence of concurrent current alcohol and current tobacco use in urban areas was 20.1% and in rural areas 14%^20^, and in metropolitan China, the last 12 months alcohol use only was 14%, tobacco use only 21% and past 12 months concurrent alcohol and tobacco use 23%^21^.

Concurrent alcohol and tobacco use is associated with several sociodemographic and health indicators. Sociodemographic factors associated with concurrent alcohol and tobacco use include, level of education (lower education among young US emergency department patients^22^, and higher education among the general adult population in Turkey^23^), male sex^23–26^, middle age^1,27–29^, lower socioeconomic status^17,19,24,25,29,30^, and residence status^24^. In Thailand the poorest group had a high tobacco consumption only and a high prevalence of alcohol consumption was observed in the richest group^17^, while in Korea lower education was associated with tobacco use only and higher education was associated with alcohol use only^19^. In the Korea study having obesity decreased the odds of tobacco use only and increased the odds of alcohol use only, hypertension increased the odds of alcohol use only, hyperlipidemia increased the odds of tobacco use only but decreased the odds of alcohol use only^19^. In Hongkong, lower income, male sex and increasing age were associated with concurrent alcohol and tobacco use^31^.

Scanty information exists on how sociodemographic and health variables are related combined tobacco and alcohol use, and a more comprehensive understanding of CTHD, CT and CHD will assist to improve population health and to decrease the tobacco and alcohol societal burden. The aim of this study was to investigate CTHD, CT, and CHD among people 15 years and older in Mongolia from 2009 to 2019.

## Methods

### Participants and procedure

Cross-sectional data from three STEPS surveys in Mongolia (2009, 2013, and 2019)^32^ were analyzed; the “overall response rate was 95.0% in 2009, 97.4% in 2013 and 98.1% in 2019”^33–35^. “A multistage stratified sampling process was carried out to randomly select participants from the target population. One individual within the age range of the survey (in 2009 and 2013 15–64 years, and in 2019 15–69 years) was chosen per household^33–35^. 

Data collection followed the “WHO STEPS methodology: step 1 included administration of a structured questionnaire (sociodemographics, medical history, medication use, and health risk behaviour), and step 2 consisted of blood pressure and anthropometric measurements”^32^. Anthropometric measurements were taken using the “Somatometre-Stanley 04–116 device and GIMA electronic scales”^33–35^. Of the three blood pressure measurements using “OMRON Model M5 automatic blood pressure monitor”^33–35^; the last two readings were averaged^36^.

All methods were carried out in accordance with relevant guidelines and regulations.

### Ethics approval and consent to participate

The study protocols were approved by the Ministry of Health Medical Ethical Committee, Mongolia, and the Ethical Review Committee of Western Pacific Regional office of WHO (WRC-WPRO) and study participants provided written informed consent^33–35^.

## Measures

### Outcome variables

*Current tobacco use* was assessed with two questions, (1) “Do you currently smoke any tobacco products, such as cigarettes, cigars or pipes?” and (2) “Do you currently use any smokeless tobacco products such as *[snuff, chewing tobacco, betel]*?” and defined as current smoking tobacco products and/or current smokeless tobacco use^36^.

*Current heavy alcohol use* was assessed in 2009 with the question, “During the past 30 days, how many times did you have, for men: five or more, for women: four or more.

standard alcoholic drinks such as beer, wine, spirits or fermented mare milk or traditional vodka in a single drinking occasion?” (Number) and in 2013 and 2019, “During the past 30 days, how many times did you have six or more standard drinks in a single drinking occasion?” (Number) A standard drink was defined as any alcoholic drink containing 10 g of pure alcohol, ethanol, which was demonstrated with showcards^33–35^. *Current heavy alcohol use* was defined as any number of times heavy drinking in the past 30 days. These substance use variables were combined resulting in four groups: 1) non-current tobacco use and non-current heavy drinking, 2) current tobacco use only (= CT); 3) current heavy drinking only (= CHD), and 4) current tobacco use and current heavy drinking (= CTHD).

### Covariates

Inadequate fruit/vegetable consumption was defined as < 5 servings/day [manual], physical activity levels were classified into low, moderate and high physical activity and sedentary behaviour (≥ 8 h/day)^37^ according to the “Global Physical Activity Questionnaire”^38^, Hypertension was assessed “based on measured blood pressure (BP) (mean of the last two of three readings) defined as systolic BP ≥ 140 mm Hg and/or diastolic BP ≥ 90 mm Hg or currently on antihypertensive medication”^39^; Body Mass Index (measured: < 18.5 kg/m^2^ underweight; 18.5–24.9 kg/m^2^ normal weight; 25.0–29.9 kg/m^2^ overweight and ≥ 30 kg/m^2^ obesity)^36^.

*Sociodemographic information* included, age (“How old are you?”—in years), education (“In total, how many years have you spent at school and in full-time study (excluding pre-school)?” (years), sex (male / female as observed), marital status (never married, currently married, cohabiting, separated, divorced, widowed), residence status (urban, rural), and ethnicity (Khalkh, Kazak, Durvud, Buriad, others)^33–35^.

## Data analysis

Statistical analyses were conducted with STATA software version 15.0. To produce representative data for the targeted population, the study sample was “weighted considering the probability of selection at three levels and accounted for participant weight/ individual weight), non-response weight, and adjustment for participant's age/sex group (population weight)”^26–28^. Multinominal logistic regression was used to assess predictors of CT, CHD, and CTHD, with non-current tobacco use and non-current heavy alcohol use as a reference category, overall, by sex and by sub-sample (survey years). Missing values (< 0.2%) were not included in the analysis. *P* < 0.05 was accepted as significant. “Svy” commands in STATA were applied to adjust for sampling design, sampling weights, and stratification, and the calculation of standard errors. Taylor linearization methods were used for variance estimation in which linear approximates (ie., the estimated variance) of a nonlinear function (ie., the true variance) are derived by taking the first-order Taylor series of the approximation.

## Results

### Sample characteristics

The sample included 5,438 people (15–64 years, median age 30 years, IQR: 22–42) in 2009, 6,013 (15–69 years, median age 40 years, IQR: 30–48) in 2013, and 6,654 persons (15–69 years, median age 34 years, IQR: 24–46) in 2019. Further sociodemographic details and information about health variables are shown in Table 1 (see Table 1 and Table 2).

**Table 1:** Sample characteristics, 15 years and older individuals, Mongolia 2009, 2013, and 2019.

Variable	Study year								
	2009	2009	2009	2013	2013	2013	2019	2019	2019
	All	Male	Female	All	Male	Female	All	Male	Female
All	N = 5438	N = 2217	N = 3221	N = 6013	N = 2719	N = 3294	N = 6654	N = 2966	N = 3688
	N (%)	N (%)	N (%)	N (%)	N (%)	N (%)	N (%)	N (%)	N (%)
**Sociodemographic variables**									
Age (years*)*									
15–29	1604 (29.5)	664 (47.1)	940 (48.0)	2437 (40.5)	1131 (24.1)	1306 (23.4)	1473 (22.1)	664 (39.5)	809 (37.9)
30–44	2264 (41.6)	910 (32.5)	1354 (31.6)	2039 (33.9)	892 (40.1)	1147 (43.7)	2491 (37.1)	1157 (33.6)	1334 (33.8)
45–64 or 69^a^	1570 (28.9)	643 (20.4)	927 (20.4)	1537 (25.6)	696 (35.8)	841 (32.9)	2690 (27.6)	1145 (26.9)	1545 (28.3)
Gender									
Femal	3221 (59.2)			3294 (54.8)			3688 (55.4)		
Male	2217 (40.8)			2719 (45.2)			2966 (44.6)		
Education (in years)									
0–9	1758 (32.3)	883 (39.7)	875 (26.2)	1613 (26.8)	884 (33.5)	729 (21.0)	1868 (28.1)	1007 (29.9)	861 (19.7)
10–11	1654 (30.4)	638 (29.2)	1016 (34.5)	1899 (31.6)	891 (31.8)	1008 (31.3)	1712 (25.7)	773 (25.7)	939 (25.8)
≥ 12	2026 (37.3)	696 (31.1)	1330 (39.3)	2501 (41.6)	944 (34.7)	1557 (47.8)	3074 (46.2)	1186 (44.4)	1888 (54.4)
Ethnic group									
Other	973 (17.9)	451 (19.8)	522 (16.3)	1185 (19.7)	585 (22.5)	600 (20.5)	1005 (35.2)	508 (17.9)	497 (13.9)
Khalkh	4460 (82.1)	1763 (80.2)	2697 (83.7)	4823 (80.3)	2131 (77.5)	2692 (79.5)	5612 (64.8)	2445 (82.1)	3176 (86.1)
Residence									
Rural	2892 (53.2)	1221 (53.7)	1671 (47.9)	3020 (50.2)	1400 (54.0)	1620 (52.1)	2339 (35.2)	1146 (39.2)	1193 (34.7)
Urban	2546 (46.8)	996 (46.3)	1550 (52.1)	2993 (49.8)	1319 (46.0)	1674 (47.9)	4315 (64.8)	1820 (60.8)	2495 (65.3)
**Health variables**									
Body mass inde*x*									
Normal	2664 (55.6)	1195 (58.4)	1469 (52.7)	2814 (41.9)	1390 (46.7)	1424 (37.1)	2583 (46.2)	1232 (47.0)	1351 (45.4)
Underweight	157 (4.5)	65 (4.6)	92 (4.5)	300 (3.6)	168 (4.0)	132 (3.2)	190 (4.6)	101 (5.2)	89 (3.9)
Overweight	1704 (27.4)	650 (26.0)	1054 (28.9)	1731 (34.0)	754 (33.1)	977 (34.9)	2257 (30.8)	1027 (31.3)	1230 (30.3)
Obesity	862 (12.5)	286 (11.1)	576 (14.0)	1013 (20.5)	381 (16.2)	632 (24.8)	1441 (18.5)	564 (16.5)	877 (20.5)
Physical activity									
Low	436 (7.4)	180 (7.2)	256 (7.6)	1403 (22.6)	611 (21.8)	792 (23.4)	1957 (28.4)	853 (27.9)	1104 (28.9)
Moderate	573 (11.5)	224 (9.9)	349 (13.2)	1513 (24.4)	625 (23.0)	888 (25.8)	1226 (18.0)	461 (14.5)	765 (21.5)
High	4429 (81.0)	1813 (82.9)	2616 (79.2)	3025 (53.0)	1442 (55.2)	1583 (50.9)	3356 (53.6)	1597 (57.6)	1759 (49.6)
Sedentary behaviour	163 (4.0)	94 (5.2)	69 (2.8)	1318 (18.2)	609 (19.2)	709 (17.3)	856 (11.5)	377 (10.7)	479 (12.2)
Inadequate fruit and vegetable intake	4895 (89.2)	2030 (90.9)	2865 (87.5)	5523 (93.8)	2542 (95.3)	2981 (92.3)	5105 (79.5)	2338 (82.4)	2767 (76.7)
Hypertension	1739 (27.3)	809 (31.4)	930 (23.1)	1347 (27.4)	704 (30.3)	643 (24.6)	1922 (23.2)	930 (25.3)	992 (21.0)

^a^15–64 years in 2009 and 2013, and 15–69 years in 2019.

**Table 2:** Sample characteristics, 15 years and older individuals, Mongolia 2009–2019.

Variable	Pooled 2009–2019		
	All	Male	Female
All	N = 18,105	N = 7902	N = 10,203
	N (%)	N (%)	N (%)
**Sociodemographic variables**			
Age (years)			
15–29	5514 (37.0)	2459 (37.3)	3055 (36.6)
30–44	6794 (35.6)	2959 (35.1)	3835 (36.0)
45–64 or 69^a^	5797 (27.5)	2484 (27.6)	3313 (27.4)
Gender			
Female	10,203 (50.1)		
Male	7902 (49.9)		
Education (in years)			
0–9	5239 (27.8)	2774 (33.7)	2465 (21.9)
10–11	5265 (29.1)	2302 (29.4)	2963 (29.8)
≥ 12	7601 (43.1)	2826 (37.8)	4775 (48.3)
Ethnic group			
Other	3163 (18.1)	1544 (19.8)	1619 (16.4)
Khalkh	14,904 (81.9)	6339 (80.2)	8565 (83.6)
Residence			
Rural	8251 (45.4)	3767 (47.6)	4484 (43.3)
Urban	9854 (54.6)	4135 (52.4)	5719 (56.7)
**Health variables**			
Body mass index			
Normal	8061 (47.7)	3817 (50.2)	4244 (45.1)
Underweight	647 (4.3)	334 (4.7)	313 (3.9)
Overweight	5692 (30.7)	2431 (30.3)	3261 (31.2)
Obesity	3316 (17.3)	1431 (14.9)	2085 (19.9)
Physical activity			
Low	37,696 (20.7)	1644 (20.1)	2152 (21.3)
Moderate	3312 (18.0)	1310 (15.6)	2002 (20.4)
High	17,918 (61.3)	4852 (64.3)	5958 (58.3)
Sedentary behaviour	2337 (11.3)	1080 (11.5)	1257 (11.0)
Inadequate fruit and vegetable intake	15,523 (86.5)	6910 (88.6)	8613 (84.3)
Hypertension	5008 (25.5)	1443 (28.5)	2565 (22.6)

^a^15-64 years in 2009 and 2013, and 15–69 years in 2019.

### Trends in the prevalence of substance use variables

Trend analysis shows that the prevalence of CT increased from 14.4% in 2009 to 15.3% in 2019, but the increase was not significant. The prevalence of CHD significantly decreased from 13.3% in 2009 to 10.3% in 2019, and the prevalence of CTHD significantly decreased from 14.2% in 2009 to 9.4% in 2019. These trends were similar by sex and age group (see Table 3).

**Table 3:** Trends in the prevalence of current tobacco use only (= CT), current heavy drinking only (= CHD), current tobacco use and current heavy drinking (= CTHD) in the Mongolia STEPS survey, 2009–2019.

Variable	Study year			*p* for trend^a^
	2009	2013	2019	
	N = 5438	N = 6013	N = 6654	
	N (% 95% CI)	N (% 95% CI)	N (% 95% CI)	
**All**				
CT	747 (14.4, 12.7–16.4)	846 (15.9, 14.3–17.6)	1007 (15.3, 14.2–16.4)	0.081
CHD	754 (13.3, 11.5–15.3)	595 (12.0, 10.6–13.7)	770 (10.3, 9.8–11.8)	0.002
CTHD	713 (14.2, 12.5–16.2)	770 (11.5, 10.3–12.8)	627 (9.4, 8.5–10.3)	< 0.001
**Male**				
CT	580 (24.2, 20.9–27.9)	707 (27.4, 25.0–30.0)	842 (26.9, 24.9–29.1)	0.058
CHD	339 (14.4, 12.3–16.7)	352 (15.4, 13.5–17.5)	381 (11.5, 10.1–13.0)	0.003
CTHD	618 (25.4, 21.9–29.2)	532 (22.0, 19.9–24.4)	566 (17.3, 15.8–19.1)	< 0.001
**Female**				
CT	167 (4.4, 3.5–5.6)	139 (4.6, 3.2–6.5)	167 (4.0, 3.3–4.8)	0.122
CHD	415 (12.2, 10.1–14.7)	243 (8.7, 7.1–10.6)	389 (10.1, 8.8–11.5)	0.038
CTHD	95 (2.8, 2.0–4.0)	38 (1.0, 0.6–1.6)	61 (1.6, 1.2–2.1)	0.003
**Age (< 37 years)**				
CT	354 (13.5, 11.5–15.8)	416 (15.0, 12.1–18.3)	373 (13.8, 12.4–15.4)	0.133
CHD	364 (12.2, 10.2–14.6)	295 (9.6, 8.0–11.5)	299 (9.9, 8.5–11.4)	0.005
CTHD	346 (12.9, 10.9–15.3)	321 (11.4, 10.1–12.8)	244 (8.0, 6.8–9.3)	< 0.001
**Age (≥ 37 years)**				
CT	393 (16.1, 13.9–18.6)	380 (16.5, 14.9–18.2)	636 (17.3, 15.8–18.8)	0.357
CHD	390 (15.3, 13.2–17.8)	300 (13.6, 11.7–15.8)	471 (12.0, 11.8–13.3)	0.010
CTHD	367 (16.6, 14.7–48.8)	249 (11.5, 9.9–12.6)	383 (11.2, 9.9–12.6)	< 0.001

^a^Trends were tested using multivariable logistic regression adjusted for sociodemographic and health variables.

### Distribution of substance use variables

In 2009, the prevalence of CT was 14.4% (24.2% among men and 4.4% among women), the prevalence of CHD was 13.3% (14.4% among men and 12.2% among women, and the prevalence of CTHD was 14.2% (25.4% among men and 2.8% among women (see Table 4) .

**Table 4:** Distribution in percent of prevalence of current tobacco use only (= CT), current heavy drinking only (= CHD), and current tobacco use and current heavy drinking (= CTHD) in the Mongolia STEPS survey, 2009.

**Variable**	Study year 2009								
	All			Male			Female		
	CT	CHD	CTHD	CT	CHD	CTHD	CT	CHD	CTHD
All	14.4	13.3	14.2	24.2	14.4	25.4	4.4	12.2	2.8
**Sociodemographic variables**									
Age (years)									
15–29	13.0	10.4	10.0	22.4	11.0	17.5	3.5	9.9	2.5
30–44	15.0	17.3	19.7	24.6	17.7	35.9	4.8	16.9	2.7
45–64 or 69	17.1	13.7	15.4	27.9	16.9	26.8	6.1	10.4	3.9
Gender									
Female	4.4	12.2	2.8						
Male	24.2	14.4	25.4						
Education (in years)									
0–9	20.4	9.4	15.4	30.5	10.8	23.1	4.7	7.1	3.3
10–11	13.3	10.9	12.9	22.6	13.2	24.0	5.2	8.8	3.3
≥ 12	9.9	19.2	14.4	17.7	20.0	29.6	3.5	18.6	2.1
Ethnic group									
Other	12.7	12.5	14.4	20.1	13.9	23.6	3.4	10.8	2.9
Khalkh	14.8	13.5	14.2	25.3	14.5	25.9	4.6	12.5	2.8
Residence									
Rural	15.0	13.1	14.0	25.3	13.8	24.2	3.2	12.4	2.3
Urban	13.8	13.5	14.5	23.0	15.0	26.8	5.6	12.1	3.3
**Health variables**									
Body mass index									
Normal	15.9	11.0	14.3	25.8	11.3	23.9	4.6	10.6	3.3
Underweight	16.1	4.1	7.5	24.6	0.0	14.6	7.1	8.4	0.0
Overweight	12.9	16.8	15.5	22.3	20.3	28.8	4.1	13.5	3.2
Obesity	11.6	19.7	13.9	21.3	22.6	29.3	3.8	17.4	1.4
Physical activity									
Low	14.1	14.8	13.8	22.4	16.9	25.2	6.0	12.8	2.8
Moderate	10.1	14.6	11.8	17.0	13.7	24.2	4.8	15.2	2.3
High	15.1	13.0	14.6	25.3	14.2	25.5	4.2	11.7	2.9
Sedentary behaviour	13.3	16.5	17.6	15.0	22.0	25.5	10.1	6.1	2.7
Inadequate fruit and vegetable intake	14.9	13.2	13.9	24.6	14.4	24.5	4.5	12.0	2.6
Hypertension	16.0	16.6	17.7	23.7	18.2	28.6	5.3	14.4	2.5

In 2013, the prevalence of CT was 15.9% (27.4% among men and 4.6% among women), the prevalence of CHD was 12.0% (15.4% among men and 8.7% among women, and the prevalence of CTHD was 11.5% (22.0% among men and 1.0% among women (see Table 5) .

**Table 5:** Distribution in percent of prevalence of current tobacco use only (= CT), current heavy drinking only (= CHD), and current tobacco use and current heavy drinking (= CTHD) in the Mongolia STEPS survey, 2013.

**Variable**	Study year 2013								
	All			Male			Female		
	CT	CHD	CTHD	CT	CHD	CTHD	CT	CHD	CTHD
All	15.9	12.0	11.5	27.4	15.4	22.0	4.6	8.7	1.0
**Sociodemographic variables**									
Age (years)									
15–29	13.7	7.0	7.7	24.2	8.2	14.1	3.0	5.8	1.2
30–44	16.4	13.5	13.6	28.7	16.7	27.3	5.2	10.7	1.2
45–64 or 69	16.9	13.7	11.4	28.1	18.9	21.5	4.8	8.1	0.6
Gender									
Female	4.6	8.7	1.0						
Male	27.4	15.4	22.0						
Education (in years)									
0–9	22.9	7.8	11.8	35.2	10.6	19.0	3.6	3.2	0.5
10–11	13.7	12.1	12.7	23.0	16.7	24.5	4.4	7.5	0.9
≥ 12	12.9	14.8	10.2	23.9	18.9	22.7	5.1	11.9	1.3
Ethnic group									
Other	15.8	11.3	12.1	26.0	14.5	22.3	4.8	7.9	1.1
Khalkh	15.9	12.2	11.3	27.8	15.7	22.0	4.5	8.9	1.0
Residence									
Rural	14.6	11.0	10.9	26.6	14.8	21.5	2.3	7.1	0.2
Urban	17.4	13.2	12.0	28.3	16.1	22.7	7.1	10.4	1.9
**Health variables**									
Body mass index									
Normal	20.6	8.0	13.5	34.2	9.6	23.0	3.3	6.1	1.4
Underweight	11.3	4.6	7.4	16.5	4.5	11.0	4.9	4.8	2.8
Overweight	13.8	13.3	12.3	22.9	16.6	24.7	5.0	10.2	0.5
Obesity	10.6	20.1	7.5	17.4	33.1	17.3	6.1	11.5	1.1
Physical activity									
Low	14.3	13.4	13.5	23.8	18.5	25.8	5.7	8.8	2.2
Moderate	16.0	13.5	10.8	27.7	15.3	21.9	5.7	11.8	1.0
High	16.0	10.9	10.8	28.1	14.5	20.6	3.3	7.1	0.4
Sedentary behaviour	17.6	10.5	15.8	28.1	13.1	27.4	6.2	7.7	3.1
Inadequate fruit and vegetable intake	16.1	12.1	11.6	27.5	15.4	22.1	4.4	8.8	0.9
Hypertension	13.7	17.2	14.2	20.9	22.9	25.1	4.8	10.4	1.1

In 2019, the prevalence of CT was 15.3% (26.9% among men and 4.0% among women), the prevalence of CHD was 10.3% (11.5% among men and 10.1% among women, and the prevalence of CTHD was 9.4% (17.3% among men and 1.6% among women (see Table 6) .

**Table 6:** Distribution in percent of prevalence of current tobacco use only (= CT), current heavy drinking only (= CHD), and current tobacco use and current heavy drinking (= CTHD) in the Mongolia STEPS survey, 2019.

Variable	Study year 2019								
	All			Male			Female		
	CT	CHD	CTHD	CT	CHD	CTHD	CT	CHD	CTHD
All	15.3	10.3	9.4	26.9	11.5	17.3	4.0	10.1	1.6
**Sociodemographic variables**									
Age (years)									
15–29	12.4	8.0	5.7	21.8	8.4	10.2	2.8	7.6	1.1
30–44	16.5	13.1	12.5	29.3	14.6	23.0	4.1	11.8	2.4
45–64 or 69	18.0	11.7	10.8	31.6	12.2	20.9	5.4	11.4	1.3
Gender									
Female	4.0	10.1	1.6						
Male	26.9	11.5	17.3						
Education (in years)									
0–9	22.9	7.8	11.8	31.0	10.8	19.7	3.8	6.1	1.0
10–11	13.7	12.1	12.7	27.8	10.4	14.4	3.6	7.8	1.1
≥ 12	12.9	14.8	10.2	23.7	12.5	17.5	4.2	12.6	2.0
Ethnic group									
Other	14.2	9.4	9.1	23.3	11.0	15.7	2.6	7.3	0.7
Khalkh	15.5	11.0	9.4	27.7	11.6	17.7	4.2	10.5	1.7
Residence									
Rural	13.8	11.4	9.6	24.8	13.8	17.5	1.7	8.8	0.9
Urban	16.2	10.4	9.2	28.4	9.9	17.2	5.2	10.8	2.0
**Health variables**									
Body mass index									
Normal	16.1	9.1	9.2	28.4	9.8	17.0	3.3	8.2	1.1
Underweight	12.3	4.9	6.6	19.9	4.8	10.7	2.0	5.1	1.0
Overweight	16.4	12.9	9.9	27.4	13.0	18.0	4.9	12.7	1.5
Obesity	14.1	13.9	9.9	25.1	16.1	19.3	5.2	12.1	2.2
Physical activity									
Low	14.6	10.1	9.5	25.0	12.0	18.1	4.7	8.2	1.5
Moderate	12.4	12.5	8.2	25.1	11.0	17.8	4.0	11.9	1.8
High	16.4	10.9	9.8	27.7	11.4	17.1	3.6	10.4	1.6
Sedentary behaviour	16.3	13.6	13.3	29.7	15.1	25.6	4.6	12.4	2.5
Inadequate fruit and vegetable intake	15.0	11.6	10.3	26.3	12.2	18.9	3.3	11.0	1.4
Hypertension	17.2	12.4	12.8	27.5	14.8	21.3	5.3	9.5	2.9

In the pooled sample, the prevalence of CT was 15.2% (26.3% among men and 4.3% among women), the prevalence of CHD was 11.8% (13.4% among men and 10.3% among women, and the prevalence of CTHD was 11.3% (21.0% among men and 1.8% among women (see Table 7).

**Table 7:** Distribution in percent of prevalence of current tobacco use only (= CT), current heavy drinking only (= CHD), and current tobacco use and current heavy drinking (= CTHD) in the Mongolia STEPS survey, 2009–2019.

Variable	Pooled 2009–2019								
	All			Male			Female		
	CT	CHD	CTHD	CT	CHD	CTHD	CT	CHD	CTHD
All	15.2	11.8	11.3	26.3	13.4	21.0	4.3	10.3	1.8
**Sociodemographic variables**									
Age (years)									
15–29	12.8	8.7	7.6	22.5	9.3	13.6	3.1	8.1	1.6
30–44	16.0	14.3	14.7	27.8	16.1	27.8	4.6	12.6	2.0
45–64 or 69	17.4	12.8	12.0	29.5	15.7	22.4	5.4	10.0	1.6
Gender									
Female									
Male									
Education (in years)									
0–9	21.0	8.7	13.1	32.0	10.7	20.7	4.1	5.6	1.7
10–11	14.3	10.6	10.8	24.7	13.3	20.5	4.4	8.0	1.7
≥ 12	12.2	14.7	10.5	22.3	16.0	21.7	4.3	13.8	1.9
Ethnic group									
Other	14.3	10.3	11.6	23.3	13.0	20.1	3.6	8.5	1.5
Khalkh	15.4	12.1	11.3	27.0	13.6	21.3	4.4	10.7	1.8
Residence									
Rural	14.5	11.8	11.5	25.5	14.1	21.0	2.4	9.3	1.1
Urban	15.9	11.9	11.3	27.0	12.8	21.0	5.7	11.0	2.3
**Health variables**									
Body mass index									
Normal	17.2	9.4	12.0	29.0	10.3	20.9	3.7	8.5	1.9
Underweight	13.2	4.6	7.1	20.4	3.3	11.9	4.4	6.1	1.1
Overweight	14.6	14.0	12.1	24.7	16.0	22.8	4.7	12.1	1.6
Obesity	12.4	17.2	9.9	21.9	22.8	20.9	5.2	13.0	1.6
Physical activity									
Low	14.4	11.6	11.2	24.4	14.5	21.2	5.2	8.9	1.9
Moderate	13.3	12.8	9.8	24.7	13.3	20.7	4.7	12.5	1.6
High	15.8	11.7	11.9	26.9	13.2	21.1	3.8	10.0	1.8
Sedentary behaviour	16.6	12.5	14.9	27.0	15.0	26.4	5.7	9.8	2.8
Inadequate fruit and vegetable intake	15.3	12.3	11.8	26.2	13.9	21.6	4.0	10.6	1.6
Hypertension	15.8	15.2	14.7	24.3	18.3	24.8	5.1	11.2	2.2

### Associations with current tobacco use only

In 2009, older age, male sex, belonging to the Khalkh ethnic group, and urban residence were positively associated, and higher education was negatively associated with CT. In the gender stratified analysis, among men, older age, and belonging to the Khalkh ethnic group were positively associated and higher education was negatively associated with CT, while among women middle age (30–44 years) and urban residence were positively associated with CT (see Table 8).

**Table 8:** Associations with current tobacco use only (with non-current tobacco and non-current heavy drinking as reference category) among 15 years and older individuals in Mongolia, 2009.

Variable	Study year 2009		
	All	Male	Female
	ARRR (95% CI)	ARRR (95% CI)	ARRR (95% CI)
**Sociodemographic variables**			
Age (years)			
15–29	1 (Reference)	1 (Reference)	1 (Reference)
30–44	2.05 (1.55–2.72)***	2.46 (1.74–3.49)***	1.87 (1.02–3.42)*
45–64 or 69	2.03 (1.54–2.67)***	2.17 (1.53–3.07)***	2.11 (0.99–4.51)
Gender			
Female	1 (Reference)	–	–
Male	12.30 (8.63–17.56)***	–	–
Education (in years)			
0–9	1 (Reference)	1 (Reference)	1 (Reference)
10–11	0.68 (0.53–0.88)**	0.63 (0.45–0.87)**	0.97 (0.53–1.79)
≥ 12	0.57 (0.46–0.71)***	0.58 (0.41–0.82)**	0.62 (0.34–1.12)
Ethnic group			
Other	1 (Reference)	1 (Reference)	1 (Reference)
Khalkh	1.39 (1.07–1.79*	1.69 (1.14–2.52)**	1.17 (0.48–2.86)
Residence			
Rural	1 (Reference)	1 (Reference)	1 (Reference)
Urban	1.35 (1.07–1.70)*	1.12 (0.78–1.62)	2.06 (1.28–3.33)**
**Health variables**			
Body mass index			
Normal	1 (Reference)	1 (Reference)	1 (Reference)
Underweight	0.86 (0.46–1.63)	0.74 (0.41–1.36)	1.60 (0.45–5.72)
Overweight	0.87 (0.66–1.15)	1.00 (0.70–1.44)	0.73 (0.46–1.15)
Obesity	0.78 (0.54–1.11)	0.91 (0.61–1.37)	0.55 (0.26–1.16)
Physical activity			
Low	1 (Reference)	1 (Reference)	1 (Reference)
Moderate	0.71 (0.41–1.22)	0.65 (0.28–1.48)	1.00 (0.58–1.71)
High	1.13 (0.75–1.70)	1.28 (0.68–2.38)	0.87 (0.53–1.42)
Sedentary behaviour	1.04 (0.63–1.71)	0.60 (0.30–1.20)	1.87 (0.69–5.12)
Inadequate fruit and vegetable intake	1.14 (0.84–1.54)	0.99 (0.59–1.55)	1.12 (0.52–2.42)
Hypertension	1.03 (0.85–1.24)	1.02 (0.80–1.30)	1.23 (0.81–1.87)

***p < 0.001; **p < 0.01; *p < 0.05; *ARRR* Adjusted relative risk ratio.

In 2013, older age, male sex, urban residence and sedentary behaviour were positively associated, and higher education, underweight, overweight and obesity were negatively associated with CT. In the gender stratified analysis, among men, older age was positively associated and higher education, underweight, overweight and obesity were negatively associated with CT, while among women urban residence was positively associated with CT (see Table 9).

**Table 9:** Associations with current tobacco use only (with non-current tobacco and non-current heavy drinking as reference category) among 15 years and older individuals in Mongolia, 2013.

Variable	Study year 2013		
	All	Male	Female
	ARRR (95% CI)	ARRR (95% CI)	ARRR (95% CI)
**Sociodemographic variables**			
Age (years)			
15–29	1 (Reference)	1 (Reference)	1 (Reference)
30–44	2.33 (1.72–3.16)***	2.63 (1.90–3.64)***	1.54 (0.75–3.16)
45–64 or 69	2.02 (1.35–3.01)***	2.36 (1.57–3.54)***	1.12 (0.46–2.72)
Gender			
Female	1 (Reference)	–	–
Male	14.99 (10.31–21.78)***	–	–
Education (in years)			
0–9	1 (Reference)	1 (Reference)	1 (Reference)
10–11	0.69 (0.51–0.93)*	0.64 (0.46–0.89)**	1.20 (0.48–3.01)
≥ 12	0.64 (0.49–0.84)***	0.62 (0.44–0.87)**	1.11 (0.48–2.55)
Ethnic group			
Other	1 (Reference)	1 (Reference)	1 (Reference)
Khalkh	0.97 (0.69–1.36)	1.15 (0.86–1.54)	0.62 (0.33–1.16)
Residence			
Rural	1 (Reference)	1 (Reference)	1 (Reference)
Urban	1.64 (1.21–2.22)***	1.25 (0.90–1.72)	3.32 (1.33–8.31)**
**Health variables**			
Body mass index			
Normal	1 (Reference)	1 (Reference)	1 (Reference)
Underweight	0.37 (0.21–0.66)***	0.29 (0.15–0.56)***	1.16 (0.48–1.58)
Overweight	0.67 (0.52–0.85)***	0.55 (0.42–0.72)***	1.54 (0.92–2.59)
Obesity	0.59 (0.44–0.80)***	0.44 (0.28–0.68)***	1.76 (0.96–3.28)
Physical activity			
Low	1 (Reference)	1 (Reference)	1 (Reference)
Moderate	1.41 (0.99–2.01)	1.30 (0.85–1.98)	1.25 (0.68–2.29)
High	1.19 (0.89–1.58)	1.22 (0.80–1.88)	0.90 (0.51–1.61)
Sedentary behaviour	1.36 (1.08–1.70)**	1.35 (0.99–1.85)	1.19 (0.76–1.86)
Inadequate fruit and vegetable intake	1.10 (0.72–1.68)	1.22 (0.74–2.03)	0.87 (0.48–1.58)
Hypertension	1.02 (0.74–1.40)	0.85 (0.60–1.20)	0.94 (0.57–1.55)

***p < 0.001; **p < 0.01; *p < 0.05; *ARRR* Adjusted relative risk ratio.

In 2019, older age, male sex, belonging to the Khalkh ethnic group, urban residence, high physical activity and sedentary behaviour were positively associated, and underweight, and obesity were negatively associated with CT. In the gender stratified analysis, among men, older age, high physical activity and sedentary behaviour were positively associated and underweight, and obesity were negatively associated with CT, while, among women, urban residence was positively associated and inadequate fruit and vegetable intake was negativeky associated with CT (see Table 10).

**Table 10:** Associations with current tobacco use only (with non-current tobacco and non-current heavy drinking as reference category) among 15 years and older individuals in Mongolia, 2019.

Variable	Study year 2019		
	All	Male	Female
	ARRR (95% CI)	ARRR (95% CI)	ARRR (95% CI)
**Sociodemographic variables**			
Age (years)			
15–29	1 (Reference)	1 (Reference)	1 (Reference)
30–44	2.12 (1.61–2.79)***	2.47 (1.78–3.41)***	1.40 (0.73–2.69)
45–64 or 69	2.38 (1.77–3.19)**	2.58 (1.85–3.59)***	1.70 (0.88–3.29)
Gender			
Female	1 (Reference)	–	–
Male	14.18 (11.08–18.16)***	–	–
Education (in years)			
0–9	1 (Reference)	1 (Reference)	1 (Reference)
10–11	0.83 (0.63–1.08)	0.82 (0.60–1.13)	0.80 (0.45–1.42)
≥ 12	0.81 (0.63–1.05)	0.74 (0.54–1.00)	1.00 (0.61–1.63)
Ethnic group			
Other	1 (Reference)	1 (Reference)	1 (Reference)
Khalkh	1.37 (1.01–1.85)*	1.40 (0.98–1.98)	1.44 (0.77–2.68)
Residence			
Rural	1 (Reference)	1 (Reference)	1 (Reference)
Urban	1.49 (1.17–1.91)**	1.23 (0.90–1.66)	2.96 (1.75–5.00)***
**Health variables**			
Body mass index			
Normal	1 (Reference)	1 (Reference)	1 (Reference)
Underweight	0.57 (0.33–0.98)*	0.47 (0.26–0.86)*	0.65 (0.15–2.87)
Overweight	0.86 (0.67–1.09)	0.82 (0.62–1.08)	1.30 (0.75–2.26)
Obesity	0.70 (0.54–0.92)**	0.67 (0.49–0.92)*	1.28 (0.76–2.14)
Physical activity			
Low	1 (Reference)	1 (Reference)	1 (Reference)
Moderate	1.07 (0.80–1.44)	1.06 (0.73–1.52)	0.86 (0.48–1.55)
High	1.36 (1.05–1.77)*	1.41 (1.03–1.93)*	0.97 (0.61–1.56)
Sedentary behaviour	1.56 (1.14–2.14)**	1.88 (1.25–2.81)**	1.13 (0.64–1.98)
Inadequate fruit and vegetable intake	0.85 (0.65–1.13)	1.07 (0.78–1.48)	0.58 (0.36–0.94)*
Hypertension	1.03 (0.85–1.24)	1.04 (0.77–1.40)	1.17 (0.75–1.82)

***p < 0.001; **p < 0.01; *p < 0.05; *ARRR* Adjusted relative risk ratio.

In the pooled sample (2009–2019), older age (45–69 years) (ARRR: 2.13, 95% CI: 1.76–2.57), male sex (ARRR: 13.18, 95% CI: 10.72–16.21), belonging to the Khalkh ethnic group (ARRR: 1.28, 95% CI: 1.07–1.53), urban residence (ARRR: 1.45, 95% CI: 1.15–1.81), high physical activity (ARRR: 1.27, 95% CI: 1.06–1.54) and sedentary behaviour (ARRR: 1.29, 95% CI: 1.06–1.58) were positively associated, and higher education (≥ 12 years) (ARRR: 0.62, 95% CI: 0.53–0.74), underweight (ARRR: 0.56, 95% CI: 0.47–0.90), overweight ARRR: 0.75, 95% CI: 0.67–0.87), and obesity (ARRR: 0.68, 95% CI: 0.57–0.81) were negatively associated with CT. In the gender stratified analysis, among men, the predictors were the same as in the overall model, while fewer predictors were found for women (see Table 11).

**Table 11:** Associations with current tobacco use only (with non-current tobacco and non-current heavy drinking as reference category) among 15 years and older individuals in Mongolia, 2009–2019.

Variable	Pooled 2009–2019		
	All	Male	Female
	ARRR (95% CI)	ARRR (95% CI)	ARRR (95% CI)
**Sociodemographic variables**			
Age (years)			
15–29	1 (Reference	1 (Reference)	1 (Reference)
30–44	2.16 (1.83–2.56)***	2.52 (2.07–3.09)***	1.59 (1.08–1.34)*
45–64 or 69	2.13 (1.76–2.57)***	2.39 (1.94–2.96)***	1.55 (0.99–2.44)
Gender			
Female	1 (Reference)	–	–
Male	13.18 (10.72–16.21)***	–	–
Education (in years)			
0–9	1 (Reference)	1 (Reference)	1 (Reference)
10–11	0.72 (0.61–0.85)***	0.71 (0.60–0.85)***	0.96 (0.66–1.41)
≥ 12	0.62 (0.53–0.74)***	0.62 (0.52–0.74)***	0.83 (0.58–1.18)
Ethnic group			
Other	1 (Reference)	1 (Reference)	1 (Reference)
Khalkh	1.28 (1.07–1.53)**	1.39 (1.14–1.70)***	0.97 (0.62–1.52)
Residence			
Rural	1 (Reference)	1 (Reference)	1 (Reference)
Urban	1.45 (1.15–1.81)**	1.26 (1.04–1.52)*	2.59 (1.77–3.80)***
**Health variables**			
Body mass index			
Normal	1 (Reference)	1 (Reference)	1 (Reference)
Underweight	0.65 (0.47–0.90)**	0.56 (0.39–0.80)**	1.20 (0.50–2.80)
Overweight	0.75 (0.67–0.87)***	0.71 (0.60–0.84)***	1.09 (0.79–1.51)
Obesity	0.68 (0.57–0.81)***	0.59 (0.48–0.74)***	1.09 (0.77–1.55)
Physical activity			
Low	1 (Reference)	1 (Reference)	1 (Reference)
Moderate	1.22 (0.96–1.52)	1.11 (0.85–1.47)	1.04 (0.72–1.48)
High	1.27 (1.06–1.54)*	1.43 (1.14–1.79)**	0.98 (0.71–1.34)
Sedentary behaviour	1.29 (1.06–1.58)**	1.42 (1.13–1.78)**	1.25 (0.89–1.76)
Inadequate fruit and vegetable intake	0.85 (0.66–1.10)	1.03 (0.80–1.33)	0.77 (0.56–1.05)
Hypertension	0.98 (0.81–1.14)	0.97 (0.82–1.16)	1.11 (0.85–1.46)

***p < 0.001; **p < 0.01; *p < 0.05; ARRR = Adjusted Relative Risk Ratio.

### Associations with current heavy drinking only

In 2009, older age, male sex, higher education, and overweight were positively associated and underweight was negativively associated with CHD. In the gender stratified analysis, among men, older age, higher education, and overweight were positively associated with CHD, while among women middle age (30–44 years), and higher education were positively associated with CHD (see Table 12).

**Table 12:** Associations with current heavy drinking only (with non-current tobacco and non-current heavy drinking as reference category) among 15 years and older individuals in Mongolia, 2009.

Variable	Study year 2009		
	All	Male	Female
	ARRR (95% CI)	ARRR (95% CI)	ARRR (95% CI)
**Sociodemographic variables**			
Age (years)			
15–29	1 (Reference)	1 (Reference)	1 (Reference)
30–44	2.02 (1.61–2.53)***	2.69 (1.74–4.15)***	1.62 (1.11–2.35)*
45–64 or 69	1.28 (0.95–1.72)	1.82 (1.20–2.74)**	0.86 (0.57–1.29)
Gender			
Female	1 (Reference)	–	–
Male	3.29 (2.55–4.25)***	–	–
Education (in years)			
0–9	1 (Reference)	1 (Reference)	1 (Reference)
10–11	1.20 (0.78–1.83)	1.11 (0.59–2.07)	1.30 (0.87–1.94)
≥ 12	2.26 (1.48–3.45)***	1.87 (1.02–3.44)*	3.12 (2.06–4.72)***
Ethnic group			
Other	1 (Reference)	1 (Reference)	1 (Reference)
Khalkh	1.23 (0.77–1.97)	1.30 (0.70–2.39)	1.15 (0.64–2.07)
Residence			
Rural	1 (Reference)	1 (Reference)	1 (Reference)
Urban	0.87 (0.61–1.26)	0.88 (0.56–1.36)	0.75 (0.48–1.18)
**Health variables**			
Body mass index			
Normal	1 (Reference)	1 (Reference)	1 (Reference)
Underweight	0.37 (0.15–0.93)*	–	0.93 (0.37–2.28)
Overweight	1.44 (1.02–2.01)*	1.84 (1.18–2.89)**	1.19 (0.82–1.75)
Obesity	1.56 (0.96–2.51)	1.81 (0.91–3.56)	1.48 (0.94–2.34)
Physical activity			
Low	1 (Reference)	1 (Reference)	1 (Reference)
Moderate	1.01 (0.55–1.83)	0.75 (0.26–2.12)	1.13 (0.58–2.19)
High	1.00 (0.66–1.50)	1.18 (0.65–2.12)	0.89 (0.48–1.62)
Sedentary behaviour	1.20 (0.57–2.51)	1.45 (0.49–4.41)	0.60 (0.13–2.76)
Inadequate fruit and vegetable intake	0.93 (0.65–1.32)	0.96 (0.53–1.73)	095 (0.66–1.38)
Hypertension	1.25 (1.00–1.58)	1.28 (0.87–1.90)	1.20 (0.83–1.73)

***p < 0.001; **p < 0.01; *p < 0.05; *ARRR* Adjusted relative risk ratio.

In 2013, older age, male sex, higher education, overweight, obesity and hypertension were positively associated with CHD. In the gender stratified analysis, among men, older age, higher education, and obesity were positively associated with CHD, while among women higher education, overweight and obesity were positively associated with CHD (see Table 13).

**Table 13:** Associations with current heavy drinking only (with non-current tobacco and non-current heavy drinking as reference category) among 15 years and older individuals in Mongolia, 2013.

Variable	Study year 2013		
	All	Male	Female
	ARRR (95% CI)	ARRR (95% CI)	ARRR (95% CI)
**Sociodemographic variables**			
Age (years)			
15–29	1 (Reference)	1 (Reference)	1 (Reference)
30–44	2.23 (1.67–2.99)***	2.85 (1.98–4.09)***	1.44 (0.99–2.09)
45–64 or 69	1.64 (1.19–2.25)**	2.42 (1.50–3.91)***	0.91 (0.55–1.49)
Gender			
Female	1 (Reference)	–	–
Male	5.91 (4.60–7.58)***	–	–
Education (in years)			
0–9	1 (Reference)	1 (Reference)	1 (Reference)
10–11	1.61 (1.10–2.35)*	1.54 (0.92–2.55)	2.20 (1.12–4.33)*
≥ 12	2.04 (1.46–2.84)***	1.64 (1.10–2.43)*	3.63 (1.70–7.76)***
Ethnic group			
Other	1 (Reference)	1 (Reference)	1 (Reference)
Khalkh	1.06 (0.71–1.58)	1.13 (0.69–1.84)	1.02 (0.62–1.69)
Residence			
Rural	1 (Reference)	1 (Reference)	1 (Reference)
Urban	1.23 (0.94–1.60)	1.05 (0.75–1.49)	1.27 (0.89–1.82)
**Health variables**			
Body mass index			
Normal	1 (Reference)	1 (Reference)	1 (Reference)
Underweight	0.53 (0.24–1.16)	0.37 (0.12–1.08)	0.83 (0.25–2.76)
Overweight	1.37 (1.04–1.82)*	1.22 (0.81–1.84)	1.56 (1.14–2.14)**
Obesity	2.17 (1.56–3.01)***	2.37 (1.53–3.66)***	1.97 (1.30–2.99)**
Physical activity			
Low	1 (Reference)	1 (Reference)	1 (Reference)
Moderate	1.05 (0.78–1.43)	0.71 (0.39–1.29)	1.53 (0.78–3.03)
High	0.83 (0.57–1.20)	0.75 (0.44–1.29)	0.97 (0.62–1.51)
Sedentary behaviour	0.72 (0.50–1.02)	0.68 (0.43–1.05)	0.71 (0.46–1.10)
Inadequate fruit and vegetable intake	1.13 (0.67–1.89)	1.02 (0.62–1.69)	1.15 (0.59–2.64)
Hypertension	1.41 (1.17–1.76)***	1.51 (1.00–2.28)*	1.26 (0.87–1.84)

***p < 0.001; **p < 0.01; *p < 0.05; *ARRR* Adjusted Relative Risk Ratio.

In 2019, older age, male sex, higher education, belonging to the Khalkh ethnic group, overweight, obesity, moderate and high physical actitivity, sedentary behaviour, inadequate fruit and vegetable intake and hypertension were positively associated and underweight was negatively associated with CHD. In the gender stratified analysis, among men, older age, higher education, sedentary behaviour, inadequate fruit and vegetable intake were positively associated and urban residence and underweight were negatively associated with CHD. Among women, older age, higher education, overweight and obesity and moderate and high physical activity were positively associated with CHD (see Table 14).

**Table 14:** Associations with current heavy drinking only (with non-current tobacco and non-current heavy drinking as reference category) among 15 years and older individuals in Mongolia, 2019.

Variable	Study year 2019		
	All	Male	Female
	ARRR (95% CI)	ARRR (95% CI)	ARRR (95% CI)
**Sociodemographic variables**			
Age (years)			
15–29	1 (Reference)	1 (Reference)	1 (Reference)
30–44	2.26 (1.66–3.08)***	3.02 (2.01–4.54)***	1.74 (1.15–2.61)**
45–64 or 69	2.08 (1.50–2.89)***	2.39 (1.54–3.70)***	1.80 (1.22–2.67)**
Gender			
Female	1 (Reference)	–	–
Male	2.49 (2.02–3.07)**	–	–
Education (in years)			
0–9	1 (Reference)	1 (Reference)	1 (Reference)
10–11	1.26 (0.93–1.70)	1.20 (0.80–1.81)	1.39 (0.88–2.17)
≥ 12	2.00 (1.55–2.58)***	1.68 (1.17–2.41)**	2.50 (1.70–3.67)***
Ethnic group			
Other	1 (Reference)	1 (Reference)	1 (Reference)
Khalkh	1.41 (1.01–1.96)*	1.44 (0.90–2.29)	1.36 (0.88–2.11)
Residence			
Rural	1 (Reference)	1 (Reference)	1 (Reference)
Urban	0.80 (0.62–1.04)	0.56 (0.39–0.81)**	1.07 (0.77–1.50)
**Health variables**			
Body mass index			
Normal	1 (Reference)	1 (Reference)	1 (Reference)
Underweight	0.39 (0.19–0.79)**	0.23 (0.08–0.64)**	0.63 (0.25–1.57)
Overweight	1.27 (1.01–1.60)*	1.05 (0.74–1.49)	1.48 1.09–2.01)*
Obesity	1.36 (1.03–1.78)*	1.21 (0.80–1.82)	1.53 (1.05–2.23)*
Physical activity			
Low	1 (Reference)	1 (Reference)	1 (Reference)
Moderate	1.35 (1.01–1.79)*	0.98 (0.62–1.56)	1.63 (1.07–2.46)*
High	1.46 (1.12–1.89)**	1.25 (0.85–1.82)	1.61 (1.11–2.33)*
Sedentary behaviour	1.69 (1.27–2.25)***	2.44 (1.56–3.81)***	1.29 (0.88–1.88)
Inadequate fruit and vegetable intake	1.48 (1.12–1.95)**	1.97 (1.29–3.01)**	1.32 (0.91–1.91)
Hypertension	1.33 (1.06–1.67)*	1.36 (0.97–1.90)	0.78 (0.56–1.08)

***p < 0.001; **p < 0.01; *p < 0.05* ARRR* Adjusted relative risk ratio.

In the pooled sample (2009–2019), older age (45–69 years) (ARRR: 1.57, 95% CI: 1.28–1.94), male sex (ARRR: 3.35, 95% CI: 2.94–3.80), higher education (≥ 12 years) (ARRR: 1.73, 95% CI: 1.40–2.16), obesity (ARRR: 1.55, 95% CI: 1.26–1.90), high physical activity (ARRR: 1.29, 95% CI: 1.07–1.55), inadequate fruit and vegetable intake (ARRR: 1.28, 95% CI: 1.03–1.60), and hypertension (ARRR: 1.22, 95% CI: 1.06–1.40) were positively associated and underweight (ARRR: 0.51, 95% CI: 0.31–0.83) was negatively associated with CHD. In gender stratified analysis, among men, inadequate fruit and vegetable intake and hypertension were positively associated with CD, while among women high physical activity was positively associated with CHD (see Table 15).

**Table 15:** Associations with current heavy drinking only (with non-current tobacco and non-current heavy drinking as reference category) among 15 years and older individuals in Mongolia, 2009–2019.

Variable	Pooled 2009–2019		
	All	Male	Female
	ARRR (95% CI)	ARRR (95% CI)	ARRR (95% CI)
**Sociodemographic variables**			
Age (years)			
15–29	1 (Reference)	1 (Reference)	1 (Reference)
30–44	2.04 (1.72–2.42)***	2.68 (2.10–3.43)***	1.52 (1.20–1.93)***
45–64 or 69	1.57 (1.28–1.94)***	2.11 (1.60–2.79)***	1.12 (0.85–1.48)
Gender			
Female	1 (Reference)	–	–
Male	3.35 (2.94–3.80)***	–	–
Education (in years)			
0–9	1 (Reference)	1 (Reference)	1 (Reference)
10–11	1.30 (1.05–1.62)*	1.26 (0.93–1.75)	1.46 (1.11–1.93)**
≥ 12	1.73 (1.40–2.16)***	1.62 (1.22–2.15)***	2.57 (2.02–3.52)***
Ethnic group			
Other	1 (Reference)	1 (Reference)	1 (Reference)
Khalkh	1.13 (0.88–1.46)	1.31 (0.96–1.79)	1.22 (0.90–1.64)
Residence			
Rural	1 (Reference)	1 (Reference)	1 (Reference)
Urban	0.86 (0.70–1.06)	0.80 (0.63–1.00)	1.06 (0.85–1.32)
**Health variables**			
Body mass index			
Normal	1 (Reference)	1 (Reference)	1 (Reference)
Underweight	0.51 (0.31–0.83)**	0.18 (0.08–0.40)***	0.77 (0.45–135)
Overweight	1.27 (1.07–1.51)**	1.28 (1.01–1.62)*	1.32 (1.08–1.60)**
Obesity	1.55 (1.26–1.90)***	1.64 (1.23–2.58)***	1.49 (1.17–1.89)***
Physical activity			
Low	1 (Reference)	1 (Reference)	1 (Reference)
Moderate	1.25 (1.02–1.54)*	0.97 (0.68–1.38)	1.57 (1.15–2.15)**
High	1.29 (1.07–1.55)**	1.26 (0.94–1.67)	1.40 (1.09–1.81)**
Sedentary behaviour	1.12 (0.88–1.43)	1.35 (0.95–1.91)	0.88 (0.67–1.15)
Inadequate fruit and vegetable intake	1.28 (1.03–1.60)*	1.57 (1.15–2.15)**	1.13 (0.87–1.47)
Hypertension	1.22 (1.06–1.40)**	1.44 (1.17–1.78)***	1.06 (0.86–1.31)

***p < 0.001; **p < 0.01; *p < 0.05; *ARRR* Adjusted relative risk ratio.

### Associations with current tobacco use and heavy drinking

In 2009, older age, and male sex were positively associated and inadequate fruit and vegetable intake was negatively associated with CTHD. In the gender stratified analysis, among men, older age was positively associated with CTHD and, among women, higher education, obesity and inadequate fruit and vegateale intake were negatively associated with CTHD (see Table 16).

**Table 16:** Associations with current tobacco use and current heavy drinking (with non-current tobacco and non-current heavy drinking as reference category) among 15 years and older individuals in Mongolia, 2009.

Variable	Study year 2009		
	All	Male	Female
	ARRR (95% CI)	ARRR (95% CI)	ARRR (95% CI)
**Sociodemographic variables**			
Age (years)			
15–29	1 (Reference)	1 (Reference)	1 (Reference)
30–44	3.27 (2.13–5.02)***	4.22 (2.65–6.74)***	1.24 (0.68–2.27)
45–64 or 69	2.20 (1.48–3.28)***	2.39 (1.56–3.67)***	2.27 (0.99–5.25)
Gender			
Female	1 (Reference)	–	–
Male	23.47 (14.38–38.30)***	–	–
Education (in years)			
0–9	1 (Reference)	1 (Reference)	1 (Reference)
10–11	0.93 (0.67–1.28)	0.88 (0.60–1.30)	0.86 (0.47–1.58)
≥ 12	1.07 (0.69–1.66)	1.28 (0.80–2.05)	0.46 (0.23–0.93)*
Ethnic group			
Other	1 (Reference)	1 (Reference)	1 (Reference)
Khalkh	1.28 (0.80–2.06)	1.38 (0.75–2.56)	0.96 (0.27–3.36)
Residence			
Rural	1 (Reference)	1 (Reference)	1 (Reference)
Urban	1.29 (0.85, 1.96)	1.04 (0.67–1.63)	1.75 (0.87–3.50)
**Health variables**			
Body mass index			
Normal	1 (Reference)	1 (Reference)	1 (Reference)
Underweight	0.70 (0.28–1.73)	0.54 (0.22–1.32)	–
Overweight	0.91 (0.64–1.27)	1.16 (0.81–1.67)	0.78 (0.34–1.73)
Obesity	0.78 (0.54–1.14)	1.06 (0.66–1.71)	0.36 (0.17–0.76)**
Physical activity			
Low	1 (Reference)	1 (Reference)	1 (Reference)
Moderate	1.07 (0.61–1.89)	0.95 (0.48–1.90)	0.91 (0.25–3.32)
High	1.29 (0.86–1.92)	1.40 (0.89–2.20)	1.01 (0.41–2.47)
Sedentary behaviour	1.04 (0.64–1.69)	0.94 (0.49–1.80)	0.86 (0.18–4.16)
Inadequate fruit and vegetable intake	0.61 (0.40–0.94)*	0.65 (0.37–1.13)	0.45 (0.20–0.97)*
Hypertension	1.06 (0.80–1.40)	1.15 (0.82–1.62)	0.78 (0.42–1.44)

***p < 0.001; **p < 0.01; *p < 0.05; *ARRR* Adjusted relative risk ratio.

In 2013, older age, male sex, and sedentary behaviour were positively associated and obesity was negatively associated with CTHD. In the gender stratified analysis, among men, older age was positively associated and underweight and obesity were negatively associated with CTHD. Among women, urban residence and sedentary behaviour were positively associated, and belonging to the Khalkh ethnic group was negatively associated with CTHD (see Table 17).

**Table 17:** Associations with current tobacco use and current heavy drinking (with non-current tobacco and non-current heavy drinking as reference category) among 15 years and older individuals in Mongolia, 2013.

Variable	Study year 2013		
	All	Male	Female
	ARRR (95% CI)	ARRR (95% CI)	ARRR (95% CI)
**Sociodemographic variables**			
Age (years)			
15–29	1 (Reference)	1 (Reference)	1 (Reference)
30–44	3.38 (2.41–4.75)***	3.85 (2.74–5.40)***	1.22 (0.43–3.47)
45–64 or 69	2.21 (1.49–3.27)***	2.64 (1.81–3.85)***	0.37 (0.11–1.28)
Gender			
Female	1 (Reference)	–	–
Male	58.99 (34.99–99.47)***	–	–
Education (in years)			
0–9	1 (Reference)	1 (Reference)	1 (Reference)
10–11	1.34 (0.92–1.94)	1.30 (0.88–1.93)	1.50 (0.18–12.56)
≥ 12	1.18 (0.80–1.76)	1.19 (0.80–1.77)	1.49 (0.24–9.25)
Ethnic group			
Other	1 (Reference)	1 (Reference)	1 (Reference)
Khalkh	0.88 (0.60–1.28)	1.00 (0.69–1.44)	0.35 (0.14–0.92)*
Residence			
Rural	1 (Reference)	1 (Reference)	1 (Reference)
Urban	1.35 (0.95–1.93)	1.11 (0.78–1.59)	13.42 (3.08–58.43)***
**Health variables**			
Body mass index			
Normal	1 (Reference)	1 (Reference)	1 (Reference)
Underweight	0.58 (0.24–1.39)	0.37 (0.15–0.87)*	3.06 (0.37–25.12)
Overweight	0.78 (0.56–1.10)	0.74 (0.52–1.06)	0.39 (0.15–1.07)
Obesity	0.49 (0.31–0.75)***	0.51 (0.32–0.80)**	0.92 (0.36–2.33)
Physical activity			
Low	1 (Reference)	1 (Reference)	1 (Reference)
Moderate	1.01 (0.68–1.52)	0.90 (0.58–1.39)	0.65 (0.27–1.55)
High	0.91 (0.65–1.29)	0.91 (0.59–1.39)	0.55 (0.24–1.27)
Sedentary behaviour	1.58 (1.16–2.16)**	1.39 (1.00, 1.92)	3.82 (1.88–7.76)***
Inadequate fruit and vegetable intake	1.24 (0.69–2.22)	1.37 (0.75–2.51)	0.57 (0.26–1.27)
Hypertension	1.26 (0.93–1.71)	1.31 (0.93–1.83)	2.00 (0.68–5.88)

***p < 0.001; **p < 0.01; *p < 0.05; *ARRR* Adjusted relative risk ratio.

In 2019, older age, male sex, sedentary behaviour and hypertension were positively associated and underweight, overweight and obesity were negatively associated with CTHD. In the gender stratified analysis, among men, older age, sedentary behaviour and inadequate fruit and vegetable intake were positively associated, and overweight and obesity were negatively associated with CTHD. Among women, hypertension was positively associated with CTHD (see Table 18).

**Table 18:** Associations with current tobacco use and current heavy drinking (with non-current tobacco and non-current heavy drinking as reference category) among 15 years and older individuals in Mongolia, 2019.

Variable	Study year 2019		
	All	Male	Female
	ARRR (95% CI)	ARRR (95% CI)	ARRR (95% CI)
**Sociodemographic variables**			
Age (years)			
15–29	1 (Reference)	1 (Reference)	1 (Reference)
30–44	3.37 (2.34–4.89)***	3.97 (2.66–5.93)***	1.68 (0.74–3.80)
45–64 or 69	2.98 (2.01–4.41)***	3.39 (2.24–5.14)***	0.97 (0.40–2.39)
Gender			
Female	1 (Reference)	–	–
Male	29.03 (20.98–40.34)***	–	–
Education (in years)			
0–9	1 (Reference)	1 (Reference)	1 (Reference)
10–11	0.77 (0.58–1.04)	0.77 (0.56–1.05)	0.85 (0.27–2.73)
≥ 12	1.16 (0.87–1.55)	1.04 (0.75–1.44)	1.84 (0.78–4.35)
Ethnic group			
Other	1 (Reference)	1 (Reference)	1 (Reference)
Khalkh	1.34 (0.96–1.87)	1.33 (0.94–1.88)	1.69 (0.60–4.74)
Residence			
Rural	1 (Reference)	1 (Reference)	1 (Reference)
Urban	1.05 (0.79–1.40)	0.92 (0.67–1.25)	1.69 (0.70–4.08)
**Health variables**			
Body mass index			
Normal	1 (Reference)	1 (Reference)	1 (Reference)
Underweight	0.39 (0.19–0.79)**	0.62 (0.28–1.35)	0.88 (0.11–6.96)
Overweight	0.74 (0.55–0.99)*	0.72 (0.52–0.98)*	1.20 (0.57–2.52)
Obesity	0.71 (0.52–0.97)*	0.68 (0.48–0.98)*	1.24 (0.58–2.67)
Physical activity			
Low	1 (Reference)	1 (Reference)	1 (Reference)
Moderate	0.97 (0.69–1.37)	0.95 (0.65–1.39)	1.00 (0.45–2.22)
High	1.25 (0.94–1.65)	1.22 (0.89–1.66)	1.66 (0.76–3.63)
Sedentary behaviour	2.34 (1.67–3.25)***	2.67 (1.76–4.04)***	2.12 (0.95–4.74)
Inadequate fruit and vegetable intake	1.25 (0.87–1.79)	1.62 (1.08–2.41)*	0.71 (0.36–1.42)
Hypertension	1.53 (1.19–1.99)***	1.32 (0.99–1.77)	2.45 (1.24–4.85)**

***p < 0.001; **p < 0.01; *p < 0.05; *ARRR* Adjusted relative risk ratio.

In the pooled sample (2009–2019), older age (45–69 years) (ARRR: 2.27, 95% CI: 1.75–2.94), male sex (ARRR: 28.46, 95% CI: 22.20–36.44), high physical activity (ARRR: 1.34, 95% CI: 1.10–1.63), sedentary behaviour (ARRR: 1.87, 95% CI: 1.37–2.05), and hypertension (ARRR: 1.29, 95% CI: 1.10–1.51) were positively associated, and underweight (ARRR: 0.51, 95% CI: 0.32–0.82) and obesity (ARRR: 0.69, 95% CI: 0.56–0.87) were negatively associated with CTD. In gender stratified analysis, among men, hypertension was positively associated and underweight and obesity negatively associated CTHD, while among women urban residence was positively associated with CTHD (see Table 19).

**Table 19:** Associations with current tobacco use and current heavy drinking (with non-current tobacco and non-current heavy drinking as reference category) among 15 years and older individuals in Mongolia, 2009–2019.

Variable	Pooled 2009–2019		
	All	Male	Female
	ARRR (95% CI)	ARRR (95% CI)	ARRR (95% CI)
**Sociodemographic variables**			
Age (years)			
15–29	1 (Reference)	1 (Reference)	1 (Reference)
30–44	3.17 (2.51–4.01)***	3.84 (2.99–4.94)***	1.20 (0.77–1.85)
45–64 or 69	2.27 (1.75–2.94)***	2.66 (2.02–3.50)***	0.94 (0.53–1.65)
Gender			
Female	1 (Reference)	–	–
Male	28.46 (22.20–36.44)***	–	–
Education (in years)			
0–9	1 (Reference)	1 (Reference)	1 (Reference)
10–11	0.98 (0.81–1.20)	0.99 (0.79–1.23)	0.89 (0.50–1.59)
≥ 12	1.08 (0.87–1.34)	1.06 (0.84–1.34)	0.82 (0.47–1.43)
Ethnic group			
Other	1 (Reference)	1 (Reference)	1 (Reference)
Khalkh	1.18 (0.92–1.51)	1.27 (0.98–1.64)	0.95 (0.45–2.03)
Residence			
Rural	1 (Reference)	1 (Reference)	1 (Reference)
Urban	1.19 (0.96–1.47)	1.06 (0.84–1.34)	2.23 (1.33–3.77)**
**Health variables**			
Body mass index			
Normal	1 (Reference)	1 (Reference)	1 (Reference)
Underweight	0.51 (0.32–0.82)**	0.49 (0.30–0.80)**	0.55 (0.13–2.35)
Overweight	0.84 (0.70–1.02)	0.83 (0.67–1.03)	0.77 (0.49–1.21)
Obesity	0.69 (0.56–0.87)***	0.69 (0.53–0.88)***	0.71 (0.43–1.19
Physical activity			
Low	1 (Reference)	1 (Reference)	1 (Reference)
Moderate	1.22 (0.96–1.54)	1.03 (0.77–1.38)	0.83 (0.47–1.48)
High	1.34 (1.10–1.63)**	1.37 (1.08–1.74)**	1.31 (0.82–2.09)
Sedentary behaviour	1.87 (1.37–2.05)***	1.72 (1.36–2.18)***	2.01 (1.27–3.17)**
Inadequate fruit and vegetable intake	0.85 (0.66–1.10)	1.17 (0.86–1.59)	062 (0.38–1.03)
Hypertension	1.29 (1.10–1.51)**	1.31 (1.09–1.57)**	1.49 (0.96–2.30)

***p < 0.001; **p < 0.01; *p < 0.05; *ARRR* Adjusted relative risk ratio.

## Discussion

The current investigation aimed to estimate for the first time trends in the proportion of CT, CHD, and CTHD in three national community-based surveys in people 15 years and older from 2009 to 2019 in Mongolia. We found that the proportion of CHD and CTHD was significantly reduced from 2009 to 2019, while CT remained unchanged. The reduction in heavy drinking in Mongolia may be attributed to the strengthening of the national alcohol prevention and control programme in 2005^40^, and the “Together for Alcohol-Free Mongolia” campaign in 2011^41^. CT remained unchanged, which may be attributed to weak tobacco demand-reduction measures, such as the increase of excise taxes on tobacco^42,43^.

Throughout the study years, the proportion of CTHD (11.3%) in Mongolia was higher than in Thailand (5.2%, concurrent daily smoking and hazardous or harmful alcohol use)^16^, and South Africa (9.6%, concurrent current tobacco use and hazardous or harmful alcohol use)^44^, and lower than in Laos (18.6%)^18^. The prevalence of CHD in this study (11.8%) was lower than in South Africa (20.3%)^44^ and in Laos (28.4%)^18^, and the proportion of CT (15.2%) in this study was higher than in Thailand (4.7%)^17^, and Korea (4.2%)^19^ and similar to Laos (15.2%)^18^ but lower than in South Africa (18.2%)^44^. In line with previous studies in Mongolia a high rate of alcohol and tobacco use was found^4–8^, calling for a need for interventions. As part of tackling non-communicable disease (NCD) risk factors, such as tobacco use and heavy alcohol use, the Mongolian Government Ministry of Health, launched 'MongPEN' (WHO’s essential NCD intervention package) in 2019 to strengthen its health system to prevent and manage NCDs, by “using legislation to tackle risk factors and improve health financing; improving evidence-based management in primary care; and strengthening mechanisms for monitoring, surveillance and capacity building”^45^.

Possible reasons for a high rate of alcohol and tobacco use in Mongolia include high licensed points of alcohol sale per person, tremendous cold weather, long-standing traditional practices of alcohol use, such as homebrewing of alcohol, drinking alcohol being associated with celebrations and social events, advertising and marketing of alcohol promote its association with festivities and celebrations^4,41^. Sociocultural drivers of CHD in Mongolia include after receiving income (49.5%), celebrations (91.0%), drinking with friends and family (87.1%), custums or traditions (60.8%)^4^. Reasons for high tobacco use may include partial lack of compliance with the WHO Framework Convention on Tobacco Control^46^, for example, no anti-tobacco mass media campaigns, no smoke free laws for indoor offices, workplaces, restaurants, pubs and bars, cigarettes have not become less affordable between 2010 and 2020 (trend average), target raise of taxes to 70% of retail price is excise tax (Mongolia: 0.383), there is no toll-free telephone quit line/help line with a live person available to discuss cessation with callers in Mongolia^4,47^, and “despite the ban of tobacco advertisement by three laws (Law on Tobacco Control, Law on Advertisement and Law on Public Radio and Television), the tobacco industry freely advertises its products via all communication channels and uses misleading messages to promote their product as not harmful to health”^48^.

Regarding sociodemographic factors, this study found that middle and/or older age, and male sex, significantly increased the odds of CT, CHD, and CTHD. Higher education decreased the odds of CT among men but increased the odds of CHD among both men and women. Urban residence increased the odds of CT and CTHD among women. Consistent with previous research^23–26^, we found that men had 28.5 times higher CTHD than women. In terms of CHD, men only had 3.4 times higher rates than women, which is similar to a 3.9 male–female ratio of higher risk drinking in Mongolia^49^. Among women the prevalence of CHD (10.3%) was the highest, followed by CT (4.3%) and CTHD (1.8%), while among men the prevalence of CT was the highest (26.3%), followed by CTHD (21.0%) and CHD (13.4%). As women’s roles change in middle-income countries, such as Mongolia, the alcohol corporations have actively marketed alcohol to women, which is important in informing alcohol policy^49^. While previous findings showed that middle-age^1,27–29^ was associated with CTHD, we found that CTHD increased with age among men but not among women. One reason for this could be that our study population did not include those 65 or 70 years and older, and previous studies also included older adults.

Some previous research^17,19,24,25,29,30^, showed that lower socioeconomic status was associated with CTHD, while in our study lower education was associated with CT among men and higher education was associated with CHD among both sexes. This result is similar to previous studies in Thailand^17^ and Korea^19^. It is possible that tobacco use may be one of the stress coping strategies among participants with lower socioeconomic status, and in those with higher education and income have better access to alcohol^19^. The finding that lower education was associated with CT seems to resonate with a study in Mongolia that the level of awareness of the health risks of tobacco smoking was lower in those with lower education^6^. Urban residence increased the odds of CT among women; however, it was non-significant for CHD. A previous study in Mongolia also found a higher rate of current tobacco smoking in urban (27.5%) than rural (18.4%) areas^6^. Possible reasons for this could be an increased “exposure to tobacco products and ads, and the desire to emulate the successful Western urban dweller that has traditionally been portrayed as a smoker”^6^.

We found that underweight decreased the odds of CT, CHD and CTHD, and obesity decreased the odds of CT and CTHD among men and increased the odds of CHD among both men and women. Hypertension was positively associated with CHD and CTHD. Consistent with former research^17,19^, we found that having obesity decreased the odds of CT and increased the odds of CHD, and hypertension increased the odds of CHD. Some previous studies^50–52^ showed that smoking decreased the odds of having obesity. However, this association may not imply causation and a reverse causation is also possible^52^. The positive association between CHD and obesity among both men and women in this study seems to be consistent with the review finding that “heavy drinking and binge drinking have been more consistently linked with adiposity”^53^. In another review the intake of spirits has been positively associated with weight gain^54^. In Mongolia among the population aged 15 years and older, the recorded alcohol per capita alcohol consumption in litres of pure alcohol by type of alcoholic beverage is 54% spirits and 44% beer^5^.

Hypertension was associated with CHD among men but not women. A systematic review found that compared with nondrinkers, men and women with heavy alcohol consumption had a significantly increased risk of hypertension^55^, and any alcohol consumption was associated with an increase in the risk for hypertension in men, and in women, there was no risk increase for consumption of 1 to 2 drinks/day and an increased risk for higher consumption levels^56^.

We found an association between sedentary behaviour and CT among men, and CTHD among both sexes, which seems to concur with previous research of “older adults who are more nicotine dependent engage in more sedentary behaviour than their less nicotine dependent counterparts”^57^. Furthermore, we found a positive association between high physical activity and CT, CHD and CTHD. Similar results were found in a review in terms of CHD, where several community-based studies found a positive relationship between physical activity and alcohol use, especially in non-alcohol dependent individuals^58^. However, regarding tobacco use, a previous review showed an inverse relationship between physical activity and smoking^59^, while we found a positive relationship between high physical activity and CT. This may be explained by the result that in our study lower education was associated with CT, assuming that people with lower education are more likely to engage in physical labour and higher physical activity. Being Khalkh by ethnicity increased the odds of CT and inadequate fruit and vegetable intake increased the odds of CHD. As a result, Khalkhs may be targeted to reduce CT, and improving the diet, including adequate fruit and vegetable intake, may reduce CHD.

Our results seem to show some differences in factors associated with CT, CHD, and CTHD, which should be taken into account when addressing the different categories of substance use, calling for an integrated intervention approach to alcohol and tobacco use^59^.

The cross-sectional repeat survey design hinders us to draw causative conclusions. Most of the data was assessed by self-report, such as tobacco and alcohol use, which may have biased some responses. Furthermore, the study did not assess electronic cigarette use; the 2019 Mongolian Global Youth Tobacco Survey found that 3.5% of students currently used electronic cigarettes^60^. Future STEPS surveys in Mongolia should include electronic cigarette use. Since this study was conducted pre-COVID-19, we cannot say anything about the impact of COVID-19 on CT and CHD, which should be subject to future research.

## Conclusion

More than one in six people aged 15 years and older in Mongolia engaged in CT and more than one in ten engaged in CHD and CTHD. Trend analyses showed that CT insignificantly increased, and CHD significantly decreased from 2009 to 2019. Several sociodemographic factors (middle and/or older age and male sex for CT, CHD and CTHD, lower education among men and urban residence among women for CT, and higher education for CHD) and health variables (lower BMI for CT and CTHD among men, higher BMI for CHD and hypertension for CHD and CTHD) were identified that can help guide public interventions.

The different prevalence and associated factor of the different categories of substance use, calling for an integrated intervention approach to alcohol and tobacco use in Mongolia. CT may be addressed by increased tobacco demand-reduction measures, such as the increase of excise taxes on tobacco. The high rate of both CT and CHD call for interventions, such as WHO’s SAFER guidance towards reducing tobacco and alcohol harms in Mongolia. In particular urban dwellers may be targeted with anti-smoking media messages, and brief interventions can target hazardous alcohol use among hypertensive patients in primary care.

## Data Availability

The data on which this analysis were based are publicly available at the World Health Organization NCD Microdata Repository: URL: https://extranet.who.int/ncdsmicrodata/index.php/catalog.
